# The Debate Goes on: New Evidence for the Role of Macronutrient Distribution on Body Weight Development: A Commentary on “Effects of Macronutrient Distribution on Weight and Related Cardiometabolic Profile in Healthy Non-Obese Chinese: A 6-month, Randomized Controlled-Feeding Trial” by Wan et al., EBioMedicine 2017, 22, 200–207

**DOI:** 10.1016/j.ebiom.2017.09.018

**Published:** 2017-09-15

**Authors:** Ibrahim Elmadfa, Alexa L. Meyer

**Affiliations:** Department of Nutritional Sciences, Faculty of Life Sciences, University of Vienna, Austria, Althanstrasse 14, 1090 Vienna, Austria

There has been an ongoing debate about the role of macronutrient composition of the diet in weight management and the potential of its modification as an approach to fight obesity. The importance of this question is obvious from the high and – in ever more parts of the World – still increasing prevalence of excessive body weight and the health impairments associated with it. Indeed, according to estimates from the World Health Organization (WHO), at the global level about 11% of men and 15% of women aged ≥ 18 years had a body mass index ≥ 30 kg/m^2^ in 2014 (World Health Organization, 2017). At the national level, prevalence is even higher in many countries also among developing and especially transition countries that are increasingly adopting a so-called Westernized food pattern. This latter is characterized by a large contribution of highly processed foods rich in simple carbohydrates in the form of sugar, and fat but poor in dietary fiber, micronutrients and secondary plant components that have been associated with beneficial health effects.

A comparable trend is also observed in China. However, contrary to many Western countries where recent nutrition trends have seen a rise in simple carbohydrate intake at the expense of fat, the transition in China went in the opposite direction, from the traditional Chinese diet high in carbohydrate from rice and low in fat, to a nutrition with about twice the energy amount contributed by fat. This development was accompanied by a marked increase in overweight and obesity.

This fact prompted a dietary intervention study investigating the effects of macronutrient composition on body weight and a number of metabolic health markers in 245 healthy Chinese adults (18–35 years) conducted at the Chinese People's Liberation Army General Hospital Beijing and the Department of Food Science and Nutrition at the Zhejiang University. Results from this study have recently been published by Wan et al. (2017) and provide interesting and convincing evidence for the benefits of moderation when it comes to fat intake. Participants were randomly assigned to one of three diets, a lower fat, higher carbohydrate diet (LF-HC, 20% of energy from fat, 66% from carbohydrate), representing the traditional Chinese diet, a moderate fat, moderate carbohydrate diet (MF-MC, 30% of energy from fat, 56% from carbohydrate), corresponding to the average diet currently consumed in China and a higher fat, lower carbohydrate diet (HF-LC, 40% of energy from fat, 46% from carbohydrate) as it has been observed in some Chinese metropolitan areas. Whereas all three diets induced a slight decline of body weight and waist circumference, the effect was more pronounced with the LF-HC diet than with the MF-MC and the HF-LC. The latter showed the weakest effect. A comparable picture was seen for lipid profile markers with a greater reduction of total and LDL cholesterol following LF-HC diet than the other two. In the HF-LC diet group, total cholesterol returned to its baseline level at the end of the study suggesting a stronger and more lasting effect of the LF-HC diet. Additionally, there were slight decreases in blood pressure and glycated serum protein levels in all three groups without significant differences between them. In turn, no significant changes were observed for fasting glucose and insulin as well as adipokines (Wan et al., 2017).

Even though the observed effects are small, the particular value of this study is that it shows an effect of macronutrient distribution in the diet on body weight and composition as well as blood lipid profile in normal-weight subjects that are relevant with regard to weight maintenance and the prevention of overweight and obesity.

While the authors speculate that the decrease in body weight seen in all diet groups may have been caused by an underestimation of baseline energy intake on which the energy content of the study diets was based, this does not explain the stronger effect of the HC-LF diet on body weight and waist circumference reduction. There is ongoing debate about the contribution of single macronutrients to body weight gain and the development of obesity but a clear answer is still lacking. Despite the popularity of diets emphasizing one particular macronutrient, overweight and obesity primarily result from excessive energy intake, regardless of the source. Moreover, the majority of studies on the effects of macronutrient composition on body weight involved overweight and obese persons, limiting conclusions on initial weight gain (Howell and Kones, 2017). Epidemiological data from the US do not reveal major differences in macronutrient distribution between overweight and normal weight persons (Yancy et al., 2014). The study by Wan et al. therefore fills an important gap in the comparison of carbohydrates and fats considering that both diets were isocaloric and potential confounders like dietary fiber in a high-carbohydrate diet or high protein in some low-carbohydrate approaches were well controlled, and its setting in a middle-income country.

The results of the present study with regards to the lipid profile are all the more remarkable as the main source of fat in the diet was soybean oil that is rich in polyunsaturated (PUFA) and monounsaturated fatty acids (MUFA) (almost 60% and 25% of total fat content, respectively, Sacks et al., 2017), which have been associated with an improved lipid profile and reduced cardiovascular risk (Briggs et al., 2017; Sacks et al., 2017). Moreover, they also do not support the reported positive effects of PUFA compared to carbohydrates on insulin sensitivity and markers of glucose metabolism (Imamura et al., 2016) and the higher risk for type 2 diabetes mellitus that has been associated with a high consumption of white rice (Hu et al., 2012).

The effects of carbohydrate-rich diets on metabolic health are influenced by the type of the carbohydrate used as replacement for fat and it is widely recognized that beneficial effects are mainly attributable to complex carbohydrates, especially from minimally processed foods that are slowly absorbed (Briggs et al., 2017). Unfortunately, Wan et al. give no exact information on the rice that supplied the major part of the carbohydrates in their intervention. However, the low amount of dietary fiber intake reported in this study (14 g/day) suggests the use of white rice that has on average a rather high glycemic index (average GI of 73 for cooked rice). However, there is a high variability between different varieties with some lying in the range of wholegrain bread (Atkinson et al., 2008). On the other hand, the use of polished rice would broadly exclude effects from any non-nutritive components contained in wholegrain cereals that have been suggested to confer health effects on their own.

This is also of importance as it has to be considered that within individual diets, a higher contribution of energy from fat is generally associated with a lower total amount of food consumed due to the higher, more than double, energy content of fat compared to carbohydrates. This can result in a lower diet diversity as was shown for Austria based on data from the Austrian Nutrition Report 2003 where a lower fat intake (as percentage of total energy intake) was associated with a higher intake of fruits and vegetables and a higher nutrient and dietary fiber density of the diet but a lower meat and sweets intake (Elmadfa and Freisling, 2005). However, a strength of the study by Wan et al. is the fact that the experimental diets only differed by their contents of rice/wheat flour and soybean oil while all other foods were contained in equal amounts. Participants were allowed to eat fruit in addition to the supplied food but were asked to maintain their consumption at the same level as before the study and to keep a daily dietary record so that a confounding effect on the observed changes can be excluded.

In summary, this study, thanks to its controlled setting and the interventional approach, provides valuable new evidence for the comparison of the metabolic effects of carbohydrates in the form of starch and fat in the form of PUFA and to a lesser degree, MUFA, in healthy normal-weight individuals, suggesting that a carbohydrate-accented diet might be a better option for weight and health maintenance than a diet with a higher fat content.

## Disclosure of Interest

The authors do not have any interest to declare nor did they receive any funding for this work.
